# The Circadian Oscillator of the Cerebellum: Triiodothyronine Regulates Clock Gene Expression in Granule Cells *in vitro* and in the Cerebellum of Neonatal Rats *in vivo*

**DOI:** 10.3389/fphys.2021.706433

**Published:** 2021-10-27

**Authors:** Tenna Bering, Henrik Hertz, Martin Fredensborg Rath

**Affiliations:** Department of Neuroscience, Faculty of Health and Medical Sciences, University of Copenhagen, Copenhagen, Denmark

**Keywords:** thyroid hormone, T3, cerebellum, clock gene, circadian, suprachiasmatic nucleus, iPRECIO programmable micropump

## Abstract

The central circadian clock resides in the suprachiasmatic nucleus (SCN) of the hypothalamus, but an SCN-dependent molecular circadian oscillator is present in the cerebellar cortex. Recent findings suggest that circadian release of corticosterone is capable of driving the circadian oscillator of the rat cerebellum. To determine if additional neuroendocrine signals act to shape cerebellar clock gene expression, we here tested the role of the thyroid hormone triiodothyronine (T3) in regulation of the cerebellar circadian oscillator. In cultured cerebellar granule cells from mixed-gender neonatal rats, T3 treatment affected transcript levels of the clock genes *Per2*, *Arntl*, *Nr1d1*, and *Dbp*, suggesting that T3 acts directly on granule cells to control the circadian oscillator. We then used two different *in vivo* protocols to test the role of T3 in adult female rats: Firstly, a single injection of T3 did not influence clock gene expression in the cerebellum. Secondly, we established a surgical rat model combining SCN lesion with a programmable micropump infusing circadian physiological levels of T3; however, rhythmic infusion of T3 did not reestablish differential clock gene expression between day and night in SCN lesioned rats. To test if the effects of T3 observed *in vitro* were related to the developmental stage, acute injections of T3 were performed in mixed-gender neonatal rats *in vivo*; this procedure significantly affected cerebellar expression of the clock genes *Per1*, *Per2*, *Nr1d1*, and *Dbp*. Developmental comparisons showed rhythmic expression of all clock genes analyzed in the cerebellum of adult rats only, whereas T3 responsiveness was limited to neonatal animals. Thus, T3 shapes cerebellar clock gene profiles in early postnatal stages, but it does not represent a systemic circadian regulatory mechanism linking the SCN to the cerebellum throughout life.

## Introduction

Basic physiology includes a number of 24 h circadian biological rhythms, including rhythmic secretion of hormones (Kalsbeek and Fliers, 2013; Challet, 2015). The circadian control in mammals emanates from the suprachiasmatic nucleus (SCN) of the hypothalamus, in which each neuron contains a molecular clock of transcriptional autoregulatory loops involving clock genes and clock gene products (Reppert and Weaver, 2002). This molecular clockwork exists in numerous tissues as so-called peripheral oscillators controlled by the master clock of the SCN (Balsalobre et al., 1998; Ko and Takahashi, 2006). In the cerebellum, rhythms of clock gene transcription are detectable in neurons of the cerebellar cortex and display a 6 h delay as compared to the SCN (Mendoza et al., 2010; Rath et al., 2012, 2014; Bering et al., 2017). Lesion studies show that an input from the SCN is a prerequisite for rhythmic clock gene expression in the cerebellum (Rath et al., 2012), and although many areas of the brain receive neural connections from the SCN, neither anterograde nor retrograde tracing studies have established a direct neural pathway between the SCN and the cerebellum (Vrang et al., 1995, 1997). In this regard, we have recently shown that rhythmic administration of the circadian hormone corticosterone can reintroduce a rhythm in rat cerebellar clock gene expression otherwise abolished by SCN ablation (Bering et al., 2020). However, in adrenalectomized rats devoid of corticosterone, local cerebellar oscillations in clock genes are still detectable, indicating that additional factors act to shape circadian signaling to the cerebellum (Bering et al., 2020). In the present study, we aim to determine whether thyroid hormone provides a signal to control clock gene expression in the cerebellum.

The interplay between thyroid hormones and the brain is evident from studies reporting crucial roles for thyroid hormones both during brain development (Legrand, 1979; Chatonnet et al., 2011) and in maintaining normal brain functions in adulthood (Smith et al., 2002; Remaud et al., 2014). The release of thyroid hormones is controlled through the hypothalamic-pituitary-thyroid axis; various degrees of circadian rhythmicity in this system have been previously reported. In the rat, low amplitude daily rhythms in serum triiodothyronine (T3) with the acrophase within the animal’s rest phase, e.g., peaking levels during the light phase, have been described (Jordan et al., 1980; Campos-Barros et al., 1997; Kalsbeek et al., 2000; Fahrenkrug et al., 2017). However, other studies have not been able to detect a circadian rhythm in T3 in rat serum (Soutto et al., 1998). The overall picture of circadian biology of T3 is further complicated by marked intra- and inter-individual variations in T3 serum concentrations obtained in studies based on blood sampling for several consecutive days (Rookh et al., 1979), as well as pronounced gender-specific differences (Kalsbeek et al., 2000). Most importantly in the context of the current study, the presence of thyroid receptors is well-documented in the rat cerebellum (Bradley et al., 1992).

With the aim of determining the possible role of thyroid hormone in regulation of the circadian oscillator of the cerebellum, we here employed both *in vitro* and surgical *in vivo* models combined with analyses of circadian clock gene expression in cerebellar neurons.

## Materials and Methods

### Animals

Female Sprague Dawley rats were obtained from Janvier Labs (Saint Berthevin Cedex, France; RRID:RGD_70508) and kept under a 12 h light/12 h dark schedule with food and water *ad libitum*. Neonatal rats (mixed gender) used in this study were 7–8 days of age and obtained from single housed pregnant mothers. All experiments on adult rats were performed on females (120–200 g at arrival), which were allowed to acclimatize for 1–2 weeks. Adult animals were housed in pairs, but single housed post-surgery. All adult animals were 8 weeks of age at the end of their experimental protocols. Surgeries were performed under sevoflurane-anesthesia to ensure fast recovery; for analgesia, the animals received carprofen, bupivacaine and buprenorphine (Bering et al., 2020); buprenorphine was administered as a single preoperative dose. Animals were killed by decapitation. All animal experiments were performed in accordance with the guidelines of EU directive 2010/63/EU. The specific experiments included in this study were approved by the Danish Council for Animal Experiments (authorization number 2017-15-0201-01190) and by The Faculty of Health and Medical Sciences at University of Copenhagen (authorization numbers P17-311 and P19-118).

### Thyroid Hormone

T3 (Sigma Aldrich; catalog number T6397) was dissolved in 0.05 N NaOH (1 mg T3/1 ml NaOH) with aliquots stored at −20°C. For each experiment, T3 aliquots were diluted in PBS. Vehicle *in vivo* groups received matching concentrations of NaOH dissolved in PBS.

### Experiments on Cerebellar Granule Cell Cultures

Rat pups were euthanized by decapitation, cerebella were dissected and primary granule cell cultures were prepared as previously described (Ilieva et al., 2019). Cells were plated in 6-well poly-l-lysine coated plates with 500,000 cells in 2 ml media per well. On day 2, 50% of the media was replaced. On day 3, the cells received either 5 μl 40 μM T3 dissolved in H_2_O for a final concentration of 100 nM or 5 μl H_2_O (control). Cells were harvested after 12 h. T3 concentration and duration of stimulation were chosen to match conditions known to affect circadian gene expression *in vitro* (Kim et al., 2010). Cells were frozen on dry ice for quantitative reverse-transcription real-time PCR (qRT-PCR).

### Triiodothyronine Delivery *in vivo* by Single Injection

For single injections of T3 in adult rats, female rats (*n* = 9; 230–257 g) received 1 ml of 25 μg/ml T3 intraperitoneally (IP) (97–109 μg T3/kg). A control group (*n* = 10; 230–261 g) received 1 ml of vehicle IP. Injections were given at Zeitgeber time (ZT) 5 and animals were euthanized at ZT9 in CO_2_ anesthesia followed by decapitation. Brains were removed and the right cerebellar hemisphere was dissected and frozen on dry ice for qRT-PCR. For single injections of T3 in neonatal rats, pups (10–13 g) received 100–130 μl 10 μg/ml T3 IP (100 μg T3/kg) on postnatal day 7 at ZT5 followed by decapitation at ZT9 or ZT17, respectively. T3 dosage and duration of stimulation were chosen to match conditions known to affect circadian gene expression *in vivo* (Klitten et al., 2008). Also, injection of T3 was chosen instead of thyroxine (T4) to avoid having endogenous deiodinase activity as a limiting and complicating factor, since only half of the endogenous T3 in the cerebellum is converted from T4 locally (Crantz et al., 1982). For each time point, T3-injected pups (*n* = 6–7) were compared to vehicle-injected controls (*n* = 5–6) from the same litter. The cerebellum was removed and frozen on dry ice for qRT-PCR.

### Suprachiasmatic Nucleus Lesion and Triiodothyronine Delivery *in vivo* by Repetitive Circadian Oscillating Infusion

Lesions of the SCN were combined with exogenous rhythmic administration of T3 by use of programmable micropumps mimicking circadian oscillations of T3 within a dose range determined by previous studies of the physiological circadian rhythm in female rats (Jordan et al., 1980; Kalsbeek et al., 2000; Fahrenkrug et al., 2017). The experimental setup consisted of three combinations of SCN lesion and pump content: Sham SCN lesioned rats with vehicle in the pump (Sham-veh), SCN lesioned rats with vehicle in the pump (SCNx-veh), and SCN lesioned rats with T3 in the pump (SCNx-T3).

**FIGURE 1:**
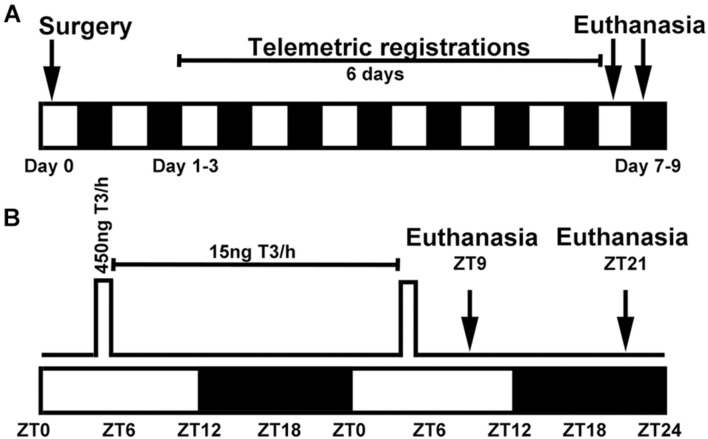
Experimental setup for surgeries and pump settings in circadian oscillating T3 infusions in rats. **(A)** Surgery was performed on day 0 and included SCN lesion and implantation of a subcutaneous telemetry transmitter and an iPRECIO pump. Telemetric registration of locomotion and body temperature was done for 6 days prior to euthanasia at either ZT9 or ZT21. The study included six experimental groups (*n* = 3–5) and a total number 26 animals; 40 animals underwent surgery, but 14 were excluded. **(B)** Pumps were programmed in 24 h repeat loops with a high T3 infusion rate of 30 μl/h from ZT4 to ZT5.5 followed by a low maintenance infusion of 1 μl/h for the remaining 22.5 h. White and black boxes indicate periods of light and darkness, respectively.

Surgeries were performed as previously described (Bering et al., 2020). In short, the SCN was ablated bilaterally with a stereotaxic electrical lesion. A telemetry transmitter (TA-F10 from Data Sciences International) was positioned subcutaneously in the midline of the back just caudal to the scapulae. Actograms of locomotor activity and body temperature were generated from a 6-day period just prior to euthanasia using ActogramJ (Schmid et al., 2011; Figure 1A). Sampling frequency was set to obtain data for 10 s every 10 min. Chi-square periodograms were used to analyze potential circadian rhythms with a predefined period of 1440 min; rhythm robustness (Qp) was calculated as a relative index for stationarity of oscillations (Refinetti, 2004). SCN lesion was verified by a loss of rhythmicity in both circadian locomotion and body temperature analyses and evaluation of Nissl-stained histological sections spanning the supraoptic area.

During the same surgery session, animals were also implanted with a programmable iPRECIO SMP-200 pump (Alzet, Tokyo, Japan) positioned subcutaneously in the midline of the back just caudal to the scapulae. Previous literature has reported that an exogenous administration of 3 μg/kg body weight T3 in thyroidectomized rats result in euthyroid T3 serum levels (Giannocco et al., 2004). All pumps were programmed to deliver repeated 24 h cycles of a high infusion rate of 30 μl/h from ZT4 to ZT5.5 followed by a low infusion rate of 1 μl/h the additional 22.5 h to avoid clotting of the tube outlet (Figure 1B). This timely profile was chosen to mimic the previously reported increase in serum T3 early in the light period (Jordan et al., 1980; Campos-Barros et al., 1997; Kalsbeek et al., 2000; Fahrenkrug et al., 2017) with a T3 half-life of 7 h (Angley et al., 1996) in the rat. The pump reservoir was prefilled with 900 μl of 15 ng/μl T3 or vehicle. Considering an individual weight variation of the rats and a prefixed T3 infusion protocol, the T3 delivery in high dose infusion was 2.8–3.6 μg/kg/1.5 h, the T3 maintenance dose range was 1.4–1.8 μg/kg/22.5 h; in all resulting in a total daily T3 dose of 4.2–5.4 μg/kg.

At the end of the experimental protocol (Figure 1), the rats were deeply anesthetized with CO_2_. From the time the rats were removed from the animal housing facility, an intracardial blood sample was collected within 3 min; animals were euthanized immediately after blood samples by decapitation at ZT9 and ZT21, respectively. Anesthesia at ZT21 was performed in dim red light. Each brain was extirpated and divided with a sagittal section 2 mm to the right of the midline. The left half of the brain including the bilateral structure of the SCN (for confirmation of SCN lesion and *in situ* hybridization) and the cerebellum dissected from the right half (for qRT-PCR) were frozen on crushed dry ice and stored at −80°C. Blood samples were placed at room temperature to clot for 30 min then centrifuged at 1,000 g for 10 min at 4°C for serum separation. Serum was stored at −80°C. Serum samples were analyzed on a Cobas 8000 (Roche Diagnostics) by Department of Clinical Biochemistry, Bispebjerg Hospital, Copenhagen, Denmark (Fahrenkrug et al., 2017).

### Quantitative Reverse-Transcription Real-Time PCR

RNA was extracted by use of TriZOL (Life Technologies) and 1 μg of extracted RNA was subjected to DNase I treatment (Thermo Fisher Scientific). cDNA synthesis from 500 ng of DNase treated RNA was performed using SuperScript III (Thermo-Fisher Scientific). All protocol steps were done according to manufacturer’s protocols. Real-time amplification was performed in a LightCycler 96 (Roche) with the program: 95°C for 10 min; 40 cycles of 95°C for 10 s, 63°C for 10 s, 72°C for 15 s. Reaction volumes of 10 μl included 0.2 μl of cDNA in a master mix consisting of 0.5 μM primer, Faststart Essential DNA Green Master (Roche) and molecular grade water. For sequences of primers used for amplification (see Table 1). Internal standard curves were generated from sequenced plasmids containing the target PCR product (Rath et al., 2013; Bering et al., 2020) in 10-fold dilutions in each run. Specificity was confirmed by melting curve analysis and gel electrophoresis. Transcript levels were normalized against those of glyceraldehyde-3-phosphate dehydrogenase (*Gapdh*).

**TABLE 1:** Sequences of primers used for qRT-PCR.

Transcript	GenBank accession number	Position	Forward primer (5′–3′)	Reverse primer (5′–3′)
*Per1*	NM_001034125.1	2411–2574	acacccagaaggaagagcaa	gcgagaacgctttgctttag
*Per2*	NM_031678.2	3319–3409	catctgccacctcagactca	ctggtgtgacttgtatcactgct
*Arntl*	NM_024362.2	2038–2132	attccagggggaaccaga	gaaggtgatgaccctcttatcct
*Clock*	NM_021856.3	2402–2483	cagccgcatccttcagtt	catggagcaaccgagatgt
*Nr1d1*	NM_001113422.2	1878–2167	gcacgaccaggtgaccctgct	gctgctccaccgaagcggaatt
*Dbp*	NM_012543.3	1131–1301	gcaaggaaagtccaggtgcccg	ctcctgccgcaatagggcgtt
*Thra*	NM_001017960.2	572–739	cctggacaaagacgagcagt	atcttgtcgatgacgcagca
*Hr*	NM_024364.3	2699–2835	tgtagcctgtggtcgcatag	ggctggagacaaactgggtc
*Gapdh*	NM_017008.4	77–386	tggtgaaggtcggtgtgaacggat	tccatggtggtgaagacgccagta

### *In situ* Hybridization

Radiochemical *in situ* hybridization was performed on coronal 12 μm cryosections of the cerebellum. DNA probes were labeled with ^35^S-dATP (Perkin Elmer) and separate sections were hybridized for each gene as previously described (Klitten et al., 2008). Probe sequences for detection of period 2 (*Per2*) and nuclear receptor subfamily 1 group D member 1 (*Nr1d1*) transcripts have been previously published (Rath et al., 2012). Hybridized sections were exposed to an X-ray film and the autoradiographical images were digitized and quantified using Scion Image Beta 4.0.2 (Scion). Optical densities of the hybridization signal were converted to dpm/mg by use of a standard curve of known values of ^14^C standards co-exposed on every X-ray film. For each animal, 4 tissue sections were measured and averaged. The area of densitometric measurement was confined to the granular layer and Purkinje cells of a dorsal folium of the vermis.

### Statistical Analyses and Experimental Design

**FIGURE 2:**
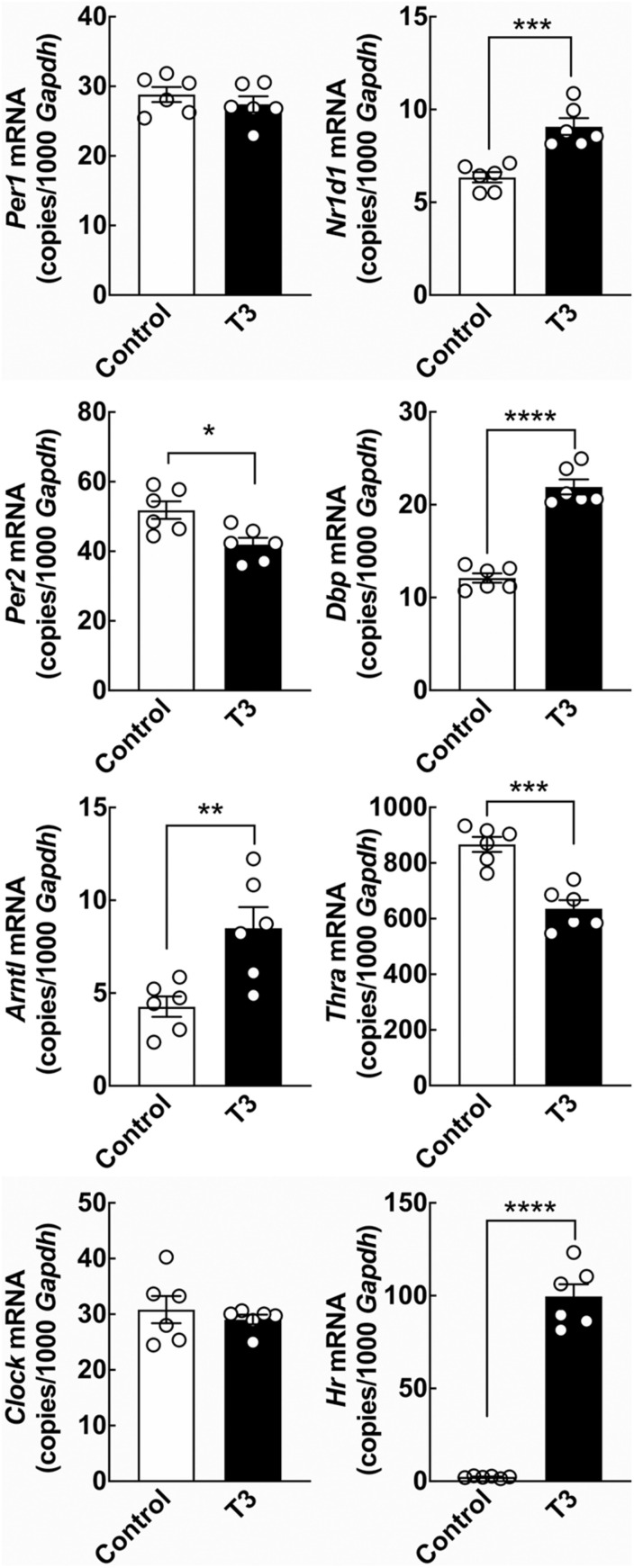
The effect of T3 on clock gene expression in cultured rat cerebellar granule cells determined by qRT-PCR. Cerebellar granule cells were exposed to 0.1 μM T3 for 12 h and compared to untreated controls. Significant effects on expression levels of the clock genes *Per2*, *Arntl*, *Nr1d1*, and *Dbp* were revealed by Student’s *t*-test. In addition significant effects on the thyroid hormone receptor *Thra* and the positive control T3-responsive gene *Hr* were detectable. Analyses of *Clock* and *Hr* included Welch’s correction. *p < 0.05; **p < 0.01; ***p < 0.001; ****p < 0.0001. *n* = 6.

**FIGURE 3:**
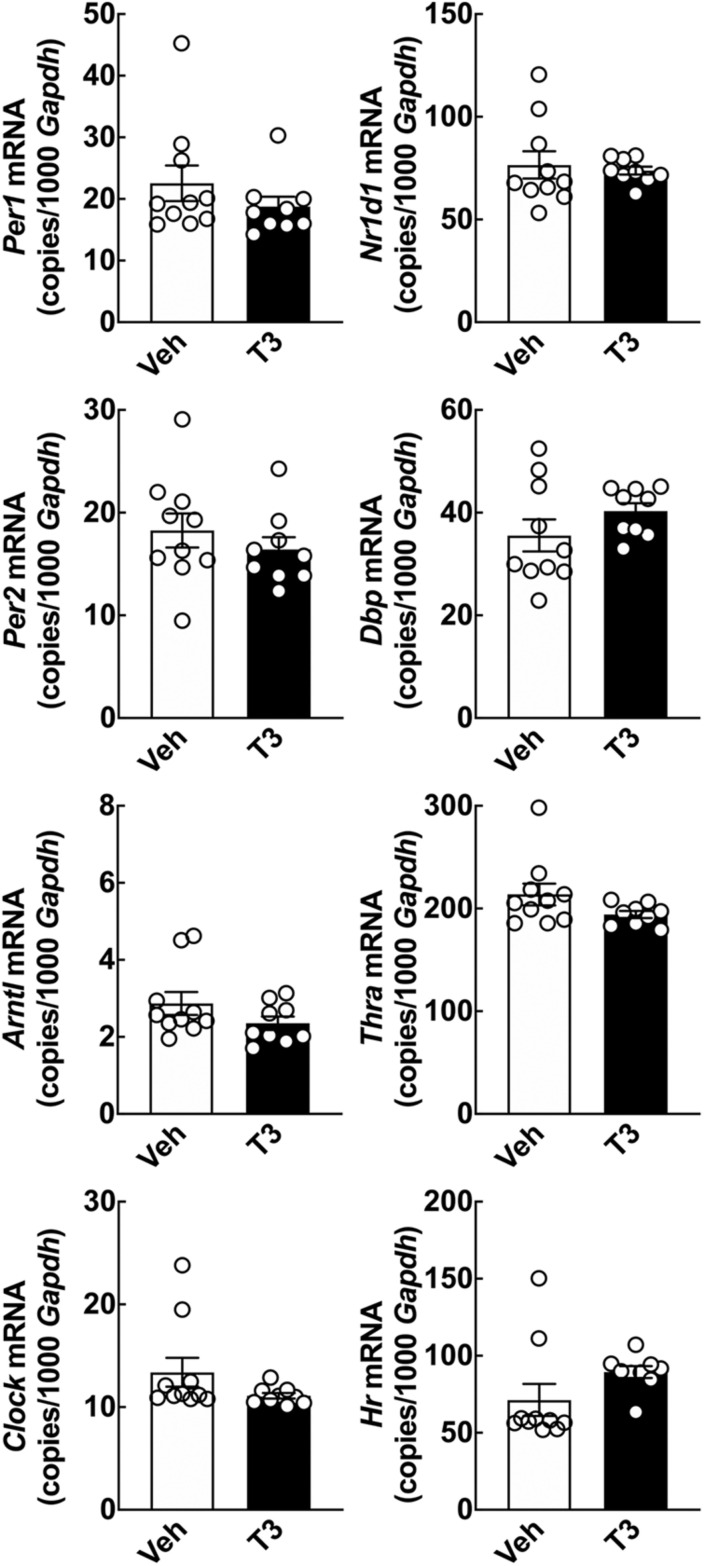
Acute effect of T3 on cerebellar clock gene expression in adult rats determined by qRT-PCR. Adult rats (8 weeks of age) received 0.1 mg/kg T3 by IP injection at ZT5 and were subsequently euthanized at ZT9. Controls received vehicle (Veh). Student’s *t*-tests did not reveal significant effects in cerebellar clock gene expression between the T3 injected rats and the vehicle group; analyses of *Nr1d1* and *Dbp* included Welch’s correction. *n* = 9–10.

**FIGURE 4:**
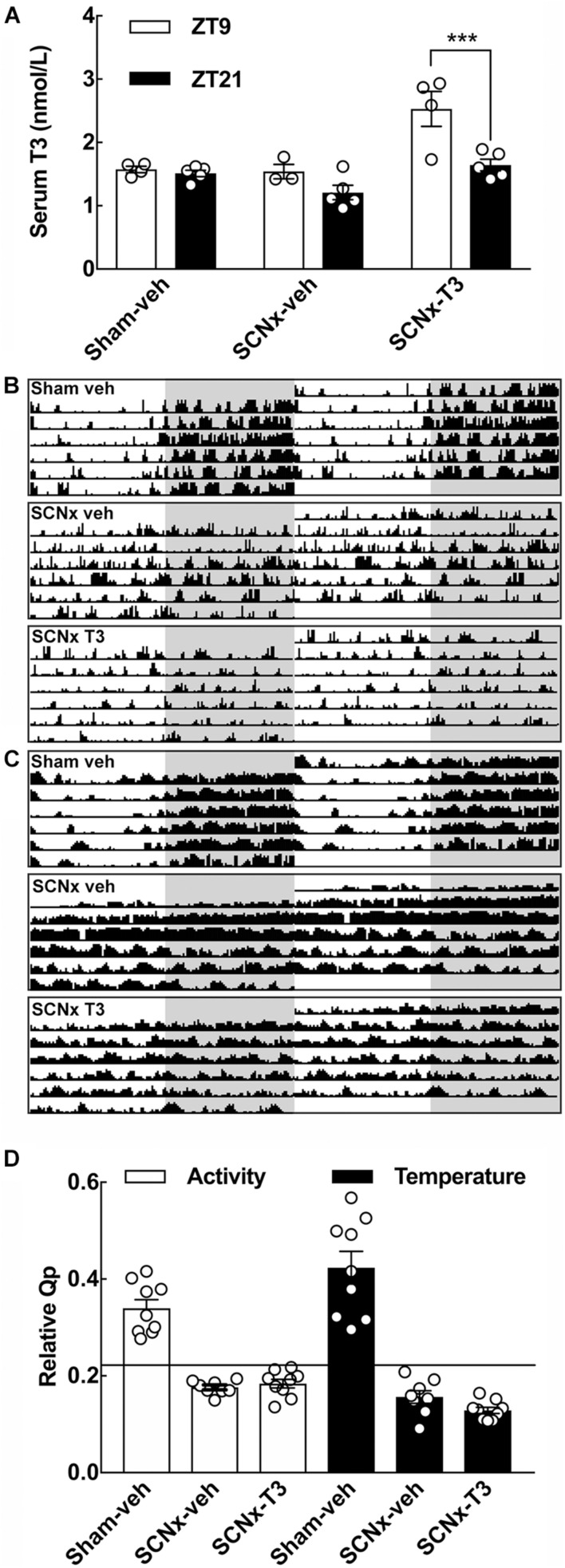
Effects of SCN-lesions and circadian oscillating T3 infusion on T3 serum concentrations, locomotion and body temperature. **(A)** Serum T3 concentrations determined from the three experimental groups of adult rats (8 weeks of age), sham-veh, SCNx-veh, and SCNx T3 at ZT9 and ZT21, respectively. Data were analyzed by two-way ANOVA followed by Bonferroni multiple-comparisons tests. A significant rhythm in serum T3 is detectable in SCNx-T3. ***p < 0.001. *n* = 3–5. **(B)** Representative double-plotted actograms of locomotor activity from each experimental group. Locomotor activity values within the range 0–25 cm/min are displayed. Shaded areas indicate the 12 h of darkness. **(C)** Representative double-plotted actograms of body temperature from each experimental group. Temperature values within the range 36–38°C are displayed. Shaded areas indicate the 12 h of darkness. **(D)** Rhythm robustness on the *y*-axis expressed as a relative *Qp*-value for activity (left) and temperature (right). Horizontal line depicts a significance level of *p* = 0.05. *n* = 8–9.

**FIGURE 5:**
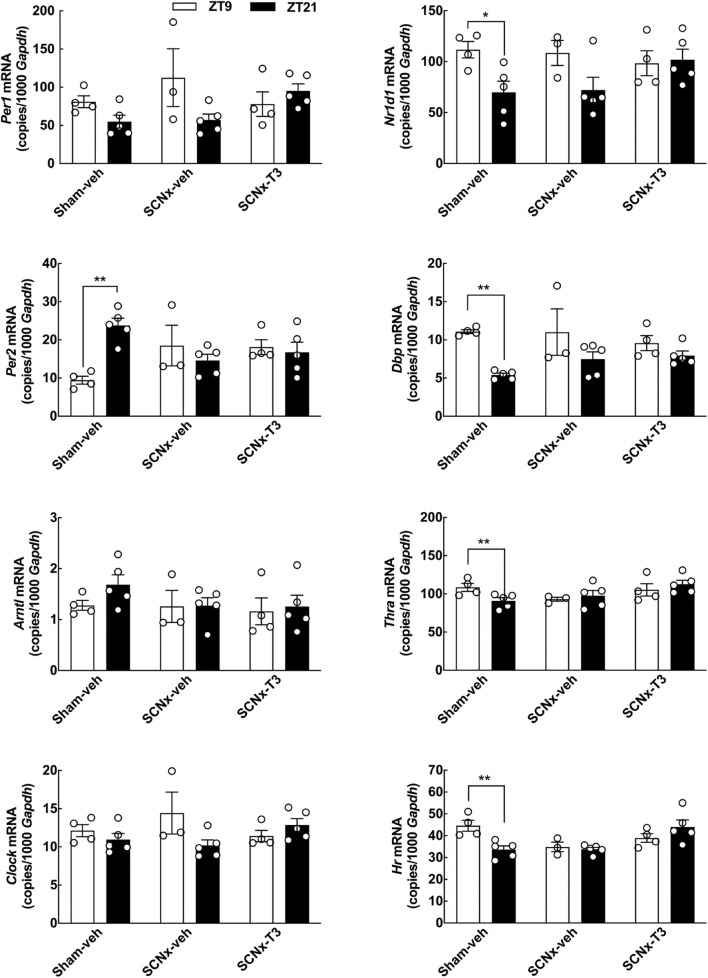
Effects of circadian oscillating T3 concentrations on clock gene expression determined by qRT-PCR. Cerebellar expression of the clock genes *Per1*, *Per2*, *Arntl, Clock*, *Nr1d1*, and *Dbp*, as well as *Thra* and *Hr*, in three experimental groups of adult rats (8 weeks of age), Sham-veh, SCNx-veh, and SCNx-T3, at ZT9, and ZT21, respectively. Quantitative data were analyzed by two-way ANOVA followed by Bonferroni multiple-comparisons tests. Day-night differences were detected in Sham-veh animals in case of the *Per2*, *Nr1d1*, *Dbp*, *Thra*, and *Hr*. Significance levels of Bonferroni multiple-comparisons tests are displayed. *p < 0.05; **p < 0.01. *n* = 3–5.

**FIGURE 6:**
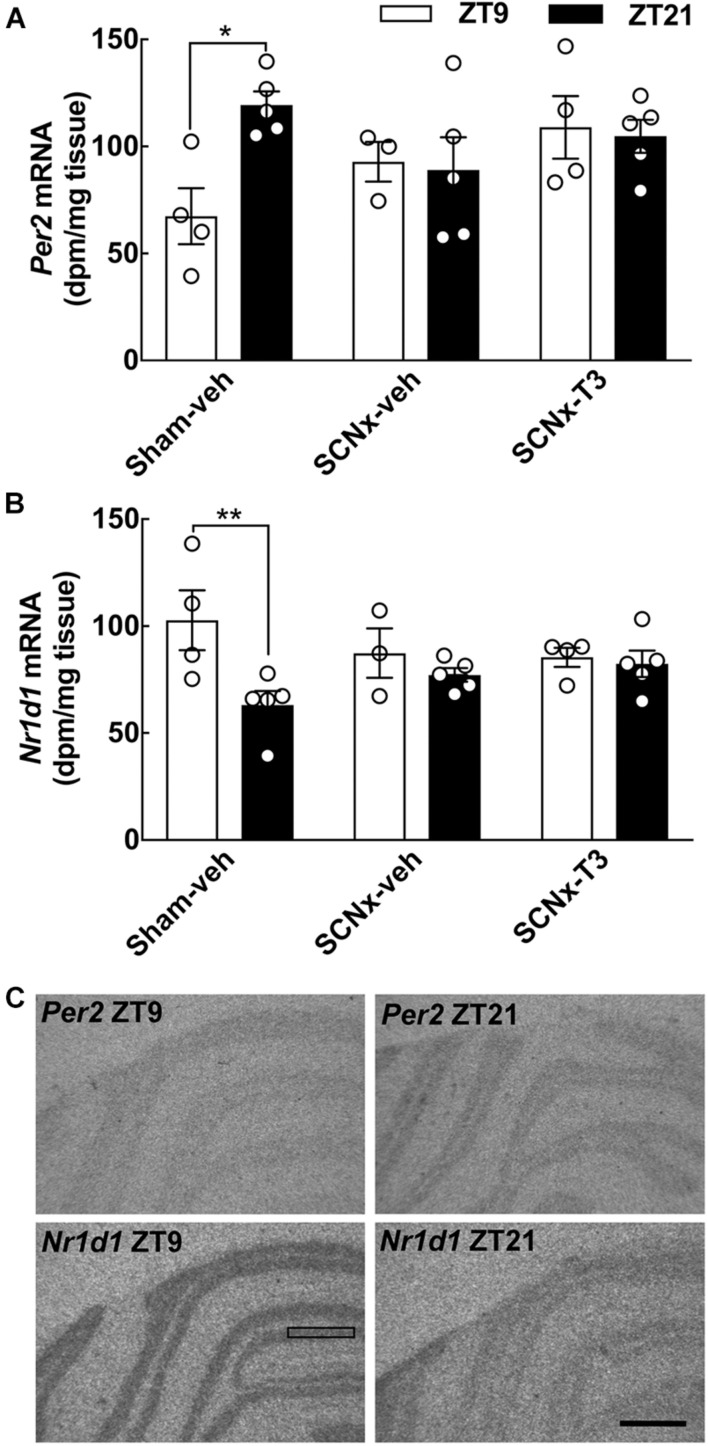
Effects of circadian oscillating T3 concentrations on clock gene expression determined by *in situ* hybridization. Radiochemical *in situ* hybridization for detection of the clock gene-encoded transcripts *Per2* **(A)** and *Nr1d1* **(B)** transcripts in the cerebellum of three experimental groups of adult rats (8 weeks of age), Sham-veh, SCNx-veh, and SCNx-T3, at ZT9, and ZT21, respectively. Representative autoradiographs are displayed **(C)**. The rectangle indicates an area of the cerebellar cortex used for quantification. Scale bar, 1 mm. Quantitative data were analyzed by two-way ANOVA followed by Bonferroni multiple-comparisons tests. Day-night differences were detected in Sham-veh animals. Significance levels of Bonferroni multiple-comparisons tests are displayed. *p < 0.05; **p < 0.01. *n* = 3–5.

**FIGURE 7:**
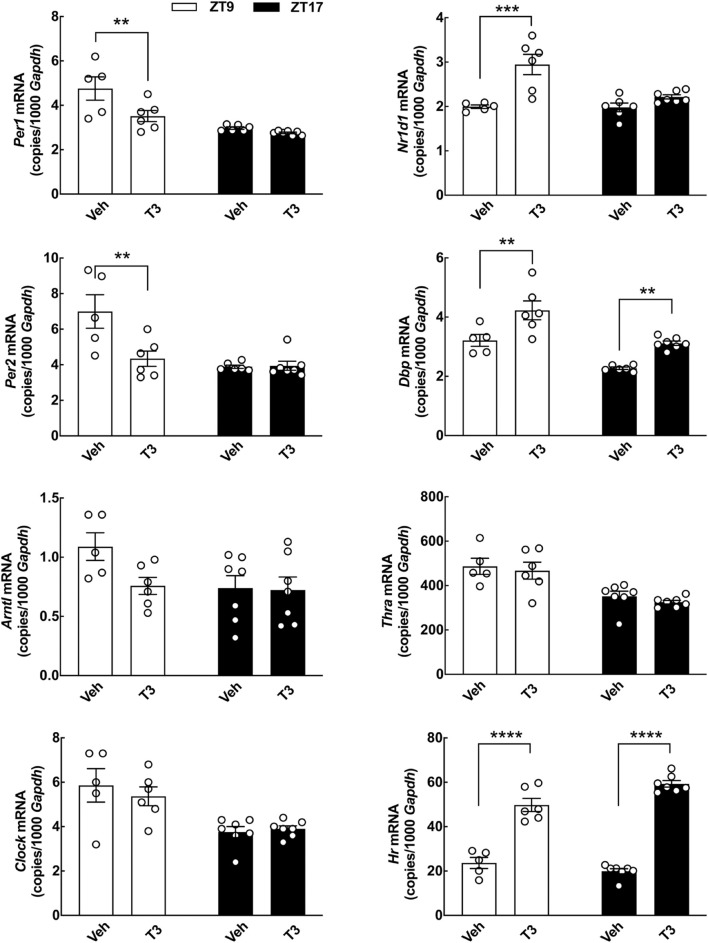
Acute effects of T3 on cerebellar clock gene expression in neonatal rats determined by qRT-PCR. Rat pups (postnatal day 7) received 0.1 mg/kg T3 by IP injection at ZT5 and were subsequently euthanized at ZT9 or ZT17. Littermate controls received vehicle (Veh). Quantitative data were analyzed by two-way ANOVA followed by Bonferroni multiple-comparisons tests. Significance levels of Bonferroni multiple-comparisons tests are displayed. **p < 0.01; ***p < 0.001; ****p < 0.0001. *n* = 5–7.

**FIGURE 8:**
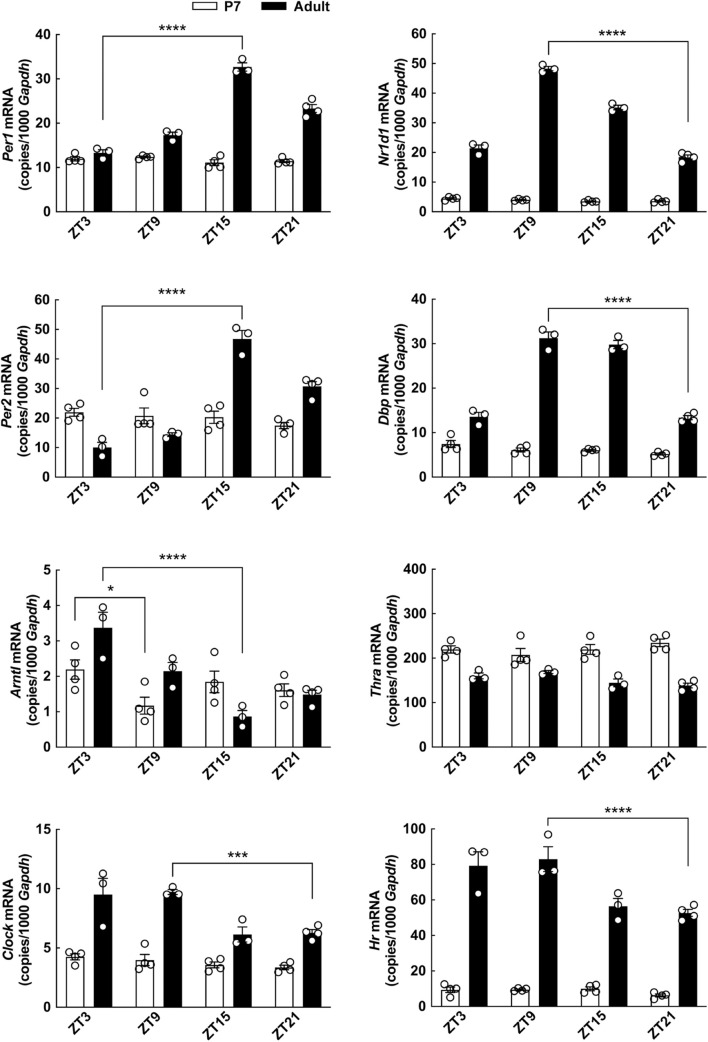
Diurnal profile of clock gene expression in the cerebellum of neonatal and adult rats determined by qRT-PCR. Rat pups (postnatal day 7) and adult rats (8 weeks of age) were kept under a 12 h light/12 h dark schedule and killed in 6-h intervals. Quantitative data were analyzed by two-way ANOVA followed by Bonferroni multiple-comparisons tests. Only significance levels of Bonferroni multiple-comparisons tests comparing the highest to the lowest expression level of each gene are displayed. *p < 0.05; ***p < 0.001; ****p < 0.0001. *n* = 3–4.

Statistical analyses were performed in Prism version 9.2 (GraphPad Software). Student’s *t*-test was used to analyze qRT-PCR data from *in vitro* studies (Figure 2) and acute effects of T3 injection in adult rats (Figure 3). *F*-test was used to assess data distribution; Welch’s correction was applied as specified in the figure legends. Two-way ANOVA followed by Bonferroni multiple-comparisons test was used for serum T3 concentrations (Figure 4), gene expression analyses in rhythmically T3 infused rats (Figures 5, 6), acute effects of T3 in neonatal rats (Figure 7) and diurnal analyses of gene expression at different developmental ages (Figure 8). *n*-values are given in figure legends and below. A *p*-value < 0.05 was considered statistically significant. Data are presented as scatter plots with group mean and SEM.

In this study, n refers to the number of individual animals or individual cell culture preparations treated as separate samples in each experimental group. In each experiment, animals from all experimental groups were tested in parallel. For blinding purposes, each sample was given a number; the number was not revealed during data acquisition, but only for the following data analysis. No data points were excluded and tests for outliers were not performed. For acute injections in adult animals (Figure 3), each experimental group contained 9–10 animals (*n* = 9–10) with a total number of 19 animals used. For acute injections in neonatal animals (Figure 6), two experiments were performed at different time points; each experiment included two experimental groups of 5–7 animals (*n* = 5–7) from the same litter with a total number of 24 animals used. For diurnal analyses of gene expression at different developmental ages (Figure 8), each experimental group included 3–4 animals (*n* = 3–4) in eight experimental groups with a total of 29 animals used. Our design for the experiment with SCN-lesions and circadian infusion of exogenous hormone (Figures 4–6) included 3–5 animals in six experimental groups (*n* = 3–5) and a total number 26 animals used. This was based on previous experiments on circadian expression of clock genes in the cerebellum (*n* = 3) (Rath et al., 2012) and the effect of SCN-lesions and exogenous hormone on cerebellar clock gene expression (*n* = 4) (Bering et al., 2020). Exclusion criteria based on telemetric and histological analyses are described above. Ten animals were excluded based on these criteria (two sham-veh, four SCNx-veh, four SCNx-T3); furthermore, four animals were euthanized post-surgery based on general welfare assessments.

## Results

### Triiodothyronine Modulates Clock Gene Expression in Cultured Cerebellar Granule Cells

To determine if T3 can act on neurons of the cerebellum to control expression of circadian clock genes, the effect of T3 stimulation on expression of selected clock genes was analyzed in cerebellar granule neurons from neonatal rats *in vitro* by qRT-PCR (Figure 2). Significant reductions in mRNA levels was found for the clock gene *Per2* (*p* < 0.05, Student’s *t*-test), whereas elevated levels of mRNA were detected for the clock genes aryl hydrocarbon receptor nuclear translocator-like protein 1 (*Arntl*) (*p* < 0.01, Student’s *t*-test), *Nr1d1* (*p* < 0.001, Student’s *t*-test) and D-box binding PAR bZIP transcription factor (*Dbp*) (*p* < 0.0001, Student’s *t*-test) as compared to controls. The transcript levels of the clock genes period 1 (*Per1*) and circadian locomotor output cycles kaput (*Clock*) were unaffected by exposure to T3. In accord with previous studies in neonatal rat cerebella (Thompson, 1996), the T3-responsive gene hairless (*Hr*), here used as a positive control, was upregulated by T3 treatment (*p* < 0.0001, Student’s *t*-test). In addition, analysis of thyroid hormone receptor alpha (*Thra*), the major thyroid hormone receptor isoform of the cerebellum (Bradley et al., 1992), revealed a reduction in transcript levels following T3-treatment (*p* < 0.001, Student’s *t*-test), in agreement with data from other *ex vivo* model systems (Hernandez-Linares et al., 2019).

### Acute *in vivo* Effects on Clock Gene Expression in the Mature Cerebellum From of a Single Triiodothyronine Injection

The alterations in clock gene expression induced by T3 in cell cultures prompted us to investigate effects of T3 *in vivo.* A single IP injection of a high dose T3 was performed at ZT5 in adult rats, which were subsequently euthanized at ZT9. qRT-PCR analyses of the cerebellum revealed no change in transcript levels of neither clock genes nor *Thra* and *Hr* 4 h after T3 injection as compared to controls (Figure 3).

### Clock Gene Expression in the Cerebellum of Suprachiasmatic Nucleus Lesioned Rats Implanted With Micropumps Releasing Exogenous Triiodothyronine in a 24 h Rhythm

To determine if a circadian rhythm in serum T3 is sufficient to drive the SCN-dependent rhythm in cerebellar clock gene expression, SCN lesions were combined with rhythmic infusion of T3 by use of a programmable micropump (Figure 1). These procedures and sham controls were combined in three surgically modified experimental groups: Sham-SCN lesioned rats with vehicle in the pump (Sham-veh), SCN lesioned rats with vehicle in the pump (SCNx-veh), and SCN lesioned rats with T3 in the pump (SCNx-T3).

T3 concentrations were determined in serum samples from rats from all experimental groups sacrificed at ZT9 and ZT21, respectively (Figure 4A). SCNx-veh animals did not differ in serum T3 concentrations between the two daily sampling time points, but a highly significant rhythm in serum T3 was detected in SCNx-T3 animals (*p* < 0.001, Bonferroni multiple-comparisons test) (Figure 4A). In the sham-veh control group, T3 serum concentrations were not significantly different between ZT9 and ZT21.

Telemetric analyses of locomotor activity and body temperature were included for verification of functional SCN ablation (Figures 4B–D). All controls (Sham-veh) exhibited circadian rhythmicity in locomotor activity with high activity levels during the dark phase, whereas lesioning the SCN (SCNx-veh) resulted in activity bursts spread randomly across the 24 h day (Figure 4B). Rhythmic exogenous administration of T3 did not restore rhythmicity in locomotion in SCN lesioned animals (SCNx-T3) (Figure 4B). Telemetric registrations of body temperature revealed similar results (Figure 4C). Hence, the controls were significantly rhythmic (*p* < 0.05) in both the Chi-square periodograms at a period length of 1440 min and in the relative *Qp*-value (Figure 4D). All SCN lesioned animals were arrhythmic in locomotor activity and body temperature irrespective the pump contents; thus, rhythmic T3 administration does not induce a circadian rhythm in these physiological outputs from the circadian system of the brain that would potentially affect gene expression in the cerebellum indirectly.

Clock gene expression levels in the cerebellum were determined with both qRT-PCR (Figure 5) and *in situ* hybridization (Figure 6) in all experimental groups. In the control group (Sham-veh), expression levels of the examined genes *Per2*, *Nr1d1*, and *Dbp* all exhibited significant differences between ZT9 and ZT21 in accordance with previous findings (Rath et al., 2012). *Per2* exhibited highest expression levels at ZT21 (qRT-PCR: *p* < 0.01; *in situ* hybridization: *p* < 0.05, Bonferroni multiple-comparisons tests), *Nr1d1* mRNA levels were highest at ZT9 (qRT-PCR: *p* < 0.05; *in situ* hybridization: *p* < 0.01, Bonferroni multiple-comparisons tests) and *Dbp* expression levels were highest at ZT9 (qRT-PCR: *p* < 0.01, Bonferroni multiple-comparisons test). In SCN lesioned rats, significant differences in cerebellar expression levels between ZT9 and ZT21 were not detected for *Per2*, *Nr1d1*, and *Dbp* neither in vehicle infused nor in T3 infused rats (Figures 5, 6), suggesting that rhythmic T3 does not restore the daily rhythm in cerebellar clock gene expression. The clock genes that did not exhibit differences in expression between ZT9 and ZT21 were not affected by rhythmic T3 infusion, neither were *Thra* and *Hr* (Figure 5).

### Acute *in vivo* Effects on Clock Gene Expression in the Neonatal Cerebellum From of a Single Triiodothyronine Injection

The apparent discrepancy between the alterations in clock gene expression induced by T3 *in vitro* and our *in vivo* data in mature rats, as well as the previously reported age-related differences in thyroid responsiveness of cerebellar gene expression (Koibuchi and Chin, 2000), prompted us to investigate the effects of T3 on clock gene expression in the cerebellum of neonatal rats *in vivo* (Figure 7). IP injections of a high dose of T3 were performed at ZT5 and the rats were subsequently euthanized at ZT9 and ZT17, respectively. qRT-PCR analyses of whole cerebella, showed a significant reduction in *Per1* and *Per2* transcripts at ZT9 (*p* < 0.01, Bonferroni multiple-comparisons tests). Elevated transcript levels were detected for *Nr1d1* at ZT9 (*p* < 0.001, Bonferroni multiple-comparisons test) and *Dbp* at both ZT9 and ZT17 (*p* < 0.01, Bonferroni multiple-comparisons tests) as compared to controls. T3 had no effect on expression of *Arntl*, *Clock*, and *Thra*; however, in contrast to our findings in adult rats (Figure 3), injections of T3 in neonatal rats lead to an increase in expression of *Hr* (Figure 7). Thus, T3 modulates the molecular circadian clock of the cerebellum *in vivo* in neonatal rats.

### Diurnal Profile of Clock Gene Expression in the Cerebellum Changes During Development

The difference in the effect of T3 on cerebellar clock gene expression at different ages prompted us to investigate the diurnal profiles of expression of clock genes, *Thra* and *Hr* in the cerebellum of neonatal and adult rats (Figure 8). Circadian rhythmic clock gene expression has previously been shown in the cerebellum of the adult rat (Rath et al., 2012), and expectedly, we here found differential expression of the clock genes *Per1*, *Per2*, *Arntl*, *Clock*, *Nr1d1*, and *Dpb* throughout the day-night cycle (*p* < 0.001, Bonferroni multiple-comparisons tests), whereas significant daily changes in the neonatal cerebellum were only detectable in case of *Arntl* (*p* < 0.05, Bonferroni multiple-comparisons test). The expression profile of all clock genes analyzed apart from *Arntl* differed significantly between ages with higher levels detected in the adult cerebella as compared to the neonatal (*p* < 0.001, two-way ANOVA analyses), suggesting that the circadian oscillator is not fully developed in the neonatal cerebellum. *Thra* expression was reduced in the adult cerebellum as compared to the neonatal (*p* < 0.0001, two-way ANOVA), whereas *Hr* was significantly increased in the adult cerebellum (*p* < 0.0001, two-way ANOVA), as indicated by previous northern blot studies (Bradley et al., 1992).

## Discussion

The cerebellar cortex has a documented SCN-dependent circadian clock gene expression profile (Rath et al., 2012), but with no traceable neural afferents from the central pacemaker in the SCN, the only known regulatory circadian signal linking the SCN to the cerebellum is rhythmic release of glucocorticoids from the adrenal glands (Bering et al., 2020). This proves the concept that crosstalk between the endocrine system and the nervous systems drives extra-hypothalamic circadian oscillators. However, in the absence glucocorticoids, rhythmic clock gene expression is still detectable in the cerebellum (Bering et al., 2020), thus prompting us to evaluate the role of another endocrine signal, i.e., T3, in regulation of a circadian brain oscillator. We here provide evidence that T3 modulates circadian clock gene expression in cerebellar granule cells of neonatal rats, but that this hormone loses its effect on the circadian clock-work of the cerebellum during development.

The present study was undertaken to determine if T3 could act as a messenger regulating the local circadian oscillator of the cerebellum. In neonatal rats and in cultured cerebellar granule cells, T3 was capable of modulating transcript levels of the clock genes *Per2*, *Nr1d1*, and *Dbp*, and under certain conditions *Per1* and *Arntl*. The *in vitro* data suggest a direct effect of T3 on granule cells at the cellular level, whereas apparent gene-specific discrepancies between individual transcripts seem to reflect variations in experimental setup and circadian profiles of individual clock genes. The immediate effects on clock gene expression in our *in vitro* experiments and *in vivo* experiments in neonatal rats, as opposed to the negative results obtained in adult rats, indicate a higher sensitivity to thyroid hormone in the developing neonate rat brain from which the cell cultures are were also prepared. This interpretation is supported by our analyses of the T3-responsive gene *Hr* (Bradley et al., 1992), which was upregulated in response to T3 in the neonatal cerebellum, whereas no effect was seen in any of our experiments performed in adult rats.

Tissues that exhibit circadian oscillations in clock genes *in vivo* will generally lose their intercellular synchrony when explanted as primary cell cultures (Nagoshi et al., 2004; Welsh et al., 2004). Serum shock can reinstall simultaneous rhythmic oscillations in clock gene transcription in culture (Balsalobre et al., 1998; Kaeffer and Pardini, 2005); this effect has been ascribed to glucocorticoids and both *in vitro* and *in vivo* studies support this conclusion (Balsalobre et al., 2000). But as shown here, another hormonal factor, i.e., T3, can exert a similar effect. From a physiological point of view, a reasonable feature in a potential distributor of a central circadian signal must be under a solid 24 h rhythmic profile. While serum corticosterone exhibits a strong circadian rhythm (Kalsbeek et al., 2012), circadian oscillations in serum T3 have been questioned with discrepancies between studies, suggestively reflecting differences in gender, as well as marked intra- and inter-individual variation (Rookh et al., 1979; Jordan et al., 1980; Campos-Barros et al., 1997; Soutto et al., 1998; Kalsbeek et al., 2000; Fahrenkrug et al., 2017). However, these previous data and the serum T3 measurements reported here do not rule out the possibility that this hormone may act as a costimulatory modulatory component in regulation of extra-hypothalamic circadian clocks. A costimulatory effect of T3 in circadian signaling has been documented in a study on mRNA expression of the circadian expression of the controlled dopamine receptor D4 gene (*Drd4*) in the rat pineal gland (Kim et al., 2010). Similarly, single injections of T3 have been reported to increase expression of otherwise circadian *Drd4* in the rat retina (Klitten et al., 2008).

In our initial *in vivo* T3 experiment on adult rats, we tested the effects of a single high dose T3 injection on clock gene expression, with time points and dose to match previous experiments (Klitten et al., 2008; Kim et al., 2010). Unlike our *in vitro* results, we did not find any *in vivo* effects on clock gene transcript levels following injection of T3 in adult rats. With the considerable sample size chosen for this experiment, it is reasonable to conclude that any immediate effects on cerebellar clock gene expression would have been detected.

Our experimental setup for testing the *in vivo* effects of T3 infused in a circadian manner was confirmed by combining analyses of telemetry, histology and serum samples; we were able to induce a significant rise in serum T3 at ZT9 by oscillating pump infusion rates in the rats with confirmed arrhythmicity in both daily locomotor activity and body temperature and complete ablation of the SCN. Since intravenous injections of T3 results in a rapid increase of nuclear T3 in the rat cerebellum (Crantz et al., 1982; Dratman et al., 1991), it is reasonable to expect that oscillations in serum T3 are converted into local oscillations in the cerebellum. On the other hand, we were not able to detect a significant difference in serum T3 in neither the matching vehicle group nor the sham animals with an intact SCN, although we had chosen female rats in our study design to take advantage of the previously reported amplitude in T3 serum concentration oscillations in females (Kalsbeek et al., 2000). As mentioned in the introduction, previous studies have demonstrated conflicting information regarding a potential circadian rhythm in T3 serum levels (van der Heide and Ende-Visser, 1980; Waner and Nyska, 1988), and our data do not support a profile with day-night differences.

Thyroid hormones are secreted by the thyroid gland primarily as the inactive T4. Within the brain, a conversion to the active T3 is primarily done by the enzyme deiodinase 2. A high concentration of this enzyme has been found in subregions of the hypothalamus (Tu et al., 1997), but it is detectable in several brain structures including the frontal cortex, hippocampus and cerebellum (Riskind et al., 1987) with a 24 h rhythm documented in the cerebellum (Campos-Barros et al., 1997; Hosoi et al., 1999; Kamiya et al., 1999; Bianco et al., 2002). Cerebellar nuclear T3 was shown to consist of 50–60% locally converted T3 from T4 (Crantz et al., 1982). In addition, circadian rhythms of T3 in brain tissue have been reported (Campos-Barros et al., 1997); thus, even if the circadian nature of circulating T3 is questionable and may be further complicated by compensatory feedback mechanisms (van Doorn et al., 1983), local hormone rhythms within the brain could still exist.

Our gene expression analyses performed using cerebellar tissue from adult rats with infusion pumps confirmed rhythmic clock gene expression in the cerebellum in accord with previous observations (Rath et al., 2012); also, SCN-lesions abolished differential clock gene expression in the cerebellum (Rath et al., 2012; Bering et al., 2020). Although we were clearly able to create a significant timely difference in T3 delivery as measured by serum concentrations, this infusion protocol did not induce day-night differences in any of the analyzed clock genes. As T3 did not influence the transcription of the clock genes in either of the two *in vivo* studies on adult rats, a role of thyroid hormone in targeting the circadian clock by receptor mediated transcriptional regulation in the mature cerebellum seems unlikely. These findings combined with the absence of a solid circadian hormone profile seem to exclude T3 from being a major circadian inducer of the cerebellar circadian oscillator.

Our developmental findings suggest that T3 influences clock gene expression at a stage where the rhythmic circadian oscillator is not yet present. Since the expression levels of the thyroid hormone receptor *Thra* are only slightly reduced in the adult cerebellum as compared to neonatal cerebellum, the lacking effect of T3 on cerebellar gene expression in the adult is striking and it does not seem to be limited to clock genes, as none of our studies on the adult cerebellum resulted in an effect of T3 on the otherwise T3-responsive gene *Hr*. The apparent conflict between our data on neonatal and adult cerebella seems to be due to an age-related mechanism, which involves other changes than the expression of the hormone receptor itself. A critical period around birth, in which genes related to cerebellar development are thyroid responsive, has been identified (Koibuchi and Chin, 2000). Since we see a clear effect of thyroid hormone on clock gene expression in granule cell cultures originating from rat pups and in pups themselves, regulation of clock gene expression by thyroid hormone seems to be possible in the aforementioned window of postnatal sensitivity to thyroid hormone. The circadian clock has been tightly linked to the cell cycle (Feillet et al., 2014; Chaix et al., 2016). Although thyroid hormone does not contribute to the timing of clock gene expression profile in the cerebellum in adult rats, it may influence cerebellar developmental processes via interaction with circadian clock components in the neonatal cerebellum, in which solid rhythmic clock gene expression, as we show here, has not been fully developed.

## Data Availability Statement

The raw data supporting the conclusions of this article will be made available by the authors, without undue reservation.

## Ethics Statement

The animal study was reviewed and approved by the Danish Council for Animal Experiments (authorization number 2017-15-0201-01190) and by The Faculty of Health and Medical Sciences at University of Copenhagen (authorization numbers P17-311 and P19-118).

## Author Contributions

MR conceived the study. TB and MR designed the experiments and wrote the manuscript. TB, HH, and MR performed the experiments and analyzed the data. HH revised the manuscript. All authors approved the final manuscript.

## Conflict of Interest

The authors declare that the research was conducted in the absence of any commercial or financial relationships that could be construed as a potential conflict of interest.

## Publisher’s Note

All claims expressed in this article are solely those of the authors and do not necessarily represent those of their affiliated organizations, or those of the publisher, the editors and the reviewers. Any product that may be evaluated in this article, or claim that may be made by its manufacturer, is not guaranteed or endorsed by the publisher.

## Abbreviations

*Arntl*: aryl hydrocarbon receptor nuclear translocator-like protein 1 (also known as *Bmal1*)
*Clock*: circadian locomotor output cycles kaput
*Dbp*: D-box binding PAR bZIP transcription factor
*Gapdh*: glyceraldehyde-3-phosphate dehydrogenase
*Hr*: hairless
IP: intraperitoneally
*Nr1d1*: nuclear receptor subfamily 1 group D member 1 (also known as *Rev-Erb α*)
*Per1*: period 1
*Per2*: period 2
qRT-PCR: quantitative reverse-transcription real-time PCR
RRID: research resource identifier
SCN: suprachiasmatic nucleus
Sham-veh: sham SCN-lesioned rats with vehicle in the pump
SCNx-T3: SCN-lesioned rats with T3 in the pump, SCNx-veh SCN-lesioned rats with vehicle in the pump
T3: triiodothyronine
T4: thyroxine
*Thra*: thyroid hormone receptor alpha
ZT: Zeitgeber time (hours after lights on).
